# Special Issue: “Advanced Light Metal and Alloys: Preparation, Characterization, and Applications”

**DOI:** 10.3390/ma16247617

**Published:** 2023-12-12

**Authors:** Pingping Liu

**Affiliations:** School of Materials Science and Engineering, University of Science and Technology Beijing, Beijing 100083, China; ppliu@ustb.edu.cn

This Special Issue presents fundamental and applied research in advanced light metal and alloys. It discusses the latest developments and innovative techniques of light metal and alloys and its practical uses.

Lightweight equipment in aerospace, automotive, and other fields has put forward new requirements for the design, characterization, preparation and comprehensive performance improvement of light metals and alloys.

Studies [1,2,3,4,5,6,7,8] report effects of alloying elements (Sm, Er, In, Bi, Zn, Mg and Cu), preparation technology (cold pressing deformation, annealing, etc.) and microstructure on performance of aluminum alloys. Martin-Raya et al. [1] discuss the rheological behavior of the A356 aluminum alloy in the semisolid state at low shear rates. Wang and Li [2] report the effect of Sm + Er and heat treatment on as-cast microstructure and mechanical properties of 7055 aluminum alloy. In this study, the cast grains of 7055 aluminum alloy were refined by adding Sm and Er, and the proper heat treatment procedure was utilized to further precipitate the rare earth phase in order to increase the alloy’s strength and toughness. Ning et al. [3] investigate the effect of initial orientation on the anisotropy in microstructure and mechanical properties of 2195 Al–Li alloy sheet during hot tensile deformation. Rečnik et al. [4] report thermodynamic and microstructural analysis of lead-free machining aluminum alloys with indium and bismuth additions. Li et al. [5] report the effect of cold pressing deformation on microstructure and residual stress of 7050 aluminum alloy die forgings. Zhu et al. [6] investigate the effects of alloying elements (Zn, Mg, and Cu) on the structural and dynamical properties of a liquid Al−9Si alloy by ab initio molecular dynamics simulations. Yi et al. [7] present an investigation of the hot stamping-in-die quenching composite forming (HFQ) process of 5083 aluminum alloy skin, which is a promising technology to compensate for the poor formability of the aluminum alloy sheet at room temperature. Gan et al. [8] investigate the influence of annealing time on microstructure and mechanical properties of Al-14.5Si alloy prepared by super-gravity solidification and cold rolling.

Studies [9,10,11] focus on the influence of friction stir welding process parameters and impurities on magnesium alloys. Mroczka et al. [9] discuss the research of friction stir welding joints of AZ91 magnesium alloy. The effect of welding forces on the microstructure and properties of AZ91 alloy is analyzed. Jung et al. [10] also investigate the tendency of having different grain structures depending on the impurity levels in AZ91 Mg-Al magnesium alloys. Khan et al. [11] discuss the effect of traverse speed variation on microstructural properties and corrosion behavior of friction stir welded WE43 Mg alloy joints.

Shi et al. [12] investigate the short-circuiting transfer, formation, and microstructure of Ti-6Al-4V alloy by external longitudinal magnetic field hybrid metal inert gas welding additive manufacturing. Cao et al. [13] analyze microstructure evolution of the Ti-46Al-8Nb-2.5V alloy during hot compression and subsequent annealing at 900 °C. Lu et al. [14,15] investigate the mechanical properties and microstructure of spiral tubes and actuators for controlled extension and retraction (STACERs) fabricated with beryllium bronze strips and Co40NiCrMo alloy. Porcaro et al. [16] describe the results obtained from an archaeometric study of a bronze Nuragic small boat model (Sardinia, Italy) dating from the Early Iron Age (presumably 9th–7th centuries BC), which may help us understand the metallurgy and manufacturing process the Final Bronze and Early Iron Ages. 

We hope that the findings presented in this Special Issue will be useful in ongoing efforts to develop new theories and novel approaches based on advanced light metal and alloys.
